# Psychiatric Disorders and Mild Cognitive Impairment in Older Veterans With Subjective Memory Complaints

**DOI:** 10.1093/geroni/igaa057.944

**Published:** 2020-12-16

**Authors:** Mia Delgadillo, Megan Frank, Aidan Boese, Tilman Schulte, J Kaci Fairchild

**Affiliations:** 1 Palo Alto University, Palo Alto, California, United States; 2 VA Palo Alto Healthcare System, Palo Alto, California, United States

## Abstract

Psychiatric disorders pose a unique risk for Alzheimer’s disease (AD). Prior research indicates psychiatric disorders in MCI increase AD vulnerability. Less research has been done to understand how psychiatric disorders may affect the development of MCI. Understanding these potentially modifiable risk factors is important as they may represent a potential target of intervention for secondary prevention of AD. The present study examines the relationship between psychiatric disorders and amnestic MCI (aMCI) in a sample of Veterans with subjective memory complaints. The sample included 150 older adults with subjective memory complaints (90% male, age = 70.6±8.2). aMCI diagnosis was based upon performance on the delayed recall trials of the Rey Auditory Verbal Learning Test and Logical Memory II of the Wechsler Memory Scale-4th edition. Psychiatric disorders (e.g., Mood Disorders, Anxiety Disorders, and Substance Use Disorders) were assessed using the Mini Neuropsychiatric Interview for DSM-IV. Logistic regression modeling demonstrated that diagnosis of anxiety disorders, but not mood or substance use disorders, was significantly associated with aMCI status. Specifically, older adults with an anxiety disorder were less likely to have aMCI than those older adults without an anxiety disorder. Additional analyses revealed that within those with aMCI (n=107), persons with a psychiatric disorder were significantly younger than those without a psychiatric disorder by an average of 6 years. These findings support prior research on the complex relationship of anxiety and cognitive impairment as well as suggest that those with psychiatric disorders may be at risk for developing aMCI at younger ages.

